# Barrier mechanisms in neonatal stroke

**DOI:** 10.3389/fnins.2014.00359

**Published:** 2014-11-07

**Authors:** Ingrid Kratzer, Sophorn Chip, Zinaida S. Vexler

**Affiliations:** Department of Neurology, University of California San FranciscoSan Francisco, CA, USA

**Keywords:** vascular permeability, microglia, leukocyte, inflammation, CSF-brain barrier, neonatal ischemia

## Abstract

Clinical data continue to reveal that the incidence of perinatal stroke is high, similar to that in the elderly. Perinatal stroke leads to significant morbidity and severe long-term neurological and cognitive deficits, including cerebral palsy. Experimental models of cerebral ischemia in neonatal rodents have shown that the pathophysiology of perinatal brain damage is multifactorial. Cerebral vasculature undergoes substantial structural and functional changes during early postnatal brain development. Thus, the state of the vasculature could affect susceptibility of the neonatal brain to cerebral ischemia. In this review, we discuss some of the most recent findings regarding the neurovascular responses of the immature brain to focal arterial stroke in relation to neuroinflammation. We also discuss a possible role of the neonatal blood-CSF barrier in modulating inflammation and the long-term effects of early neurovascular integrity after neonatal stroke on angiogenesis and neurogenesis.

## Introduction

The blood-brain barrier (BBB) protects the central nervous system (CNS) and prevents non-specific leakage of molecules and cells from the blood into the brain. Stroke can disintegrate the BBB in many ways by disrupting cell-cell communication between endothelial cells (ECs), pericytes, and astrocytes, cumulatively referred to as the “neurovascular unit” (NVU), and by affecting neurons and microglial cells, thereby enhancing injury. Reperfusion and re-oxygenation of previously ischemic brain regions further affect BBB function, restoring or disrupting, depending on various factors such as the extent, length of initial cerebral blood flow (CBF) disruption, genetic background, sex, age, and the presence of other confounding factors.

The timing of injury during brain development has a major impact on determining the pathophysiology of ischemic brain damage. Maturation of individual cell types, individual components of the NVU and of the extracellular matrix (ECM), and of individual brain regions is not synchronized, contributing to the existence of “windows of susceptibility” to ischemia during particular fetal and postnatal periods. Here we discuss some of the most recent findings regarding the neurovascular responses of the immature brain to focal arterial stroke in relation to neuroinflammation and long-term effects on repair.

## Age at the time of cerebral ischemia as a determinant of the pathophysiology of brain damage: preterm vs. term

In preterm human babies (23–32-weeks of gestation) intracerebral hemorrhage (ICH), intraventricular hemorrhage (IVH), and periventricular white matter injury (PWMI) are the most common types of ischemia-related injuries (Volpe, 2009). Clinical aspects of perinatal stroke have been extensively discussed (Ferriero, 2004; Nelson and Lynch, 2004). Vulnerability of oligodendrocyte progenitor cells (OPCs) to ischemia and hypoxia including a concomitant loss of subplate neurons, a transient neuronal subpopulation important in corticogenesis and proper wiring of the developing brain, contribute to PWMI (McQuillen et al., 2003; Volpe, 2009). The germinal matrix with its weak, leaky vasculature, high local production of vascular endothelial growth factor (VEGF), angiopoietin-2 (Angpt2), and matrix metalloproteinases (MMPs) also make the preterm brain prone to ICH and IVH (Ballabh, 2010). Low pericyte coverage and ensheathement of astrocytic endfeets along blood vessels, together with immaturity of the basal membrane (BM), also make the preterm brain susceptible to ischemia-related injury (El-Khoury et al., 2006; Braun et al., 2007). At term (between birth and 28 days of life), ischemia-related white matter injury does occur (Rothstein and Levison, 2005; Van Den Broeck et al., 2008), but injury predominantly affects gray matter (Ferriero, 2004).

Several models were developed to understand the pathophysiological mechanisms of hypoxic-ischemic encephalopathy (HIE) and focal arterial stroke at term (Yager and Ashwal, 2009). A model of hypoxia-ischemia (HI) consists of a unilateral ligation of the common carotid artery (CCA) followed by a variable duration of exposure to 8% O_2_ in postnatal day 7 (P7) rats (Rice et al., 1981), and P9 mice and mimics HIE, whereas transient middle cerebral artery occlusion (tMCAO) model in P7 rats (Derugin et al., 1998), P10 rats (Mu et al., 2003), and P9 mice (Woo et al., 2012), and a combined permanent MCAO and transient CCA occlusion in P7 rats (Renolleau et al., 1998) mimic focal arterial stroke. The HI is associated with increased CBF during systemic hypoxia whereas CBF is disrupted after MCAO. Models of HI were also developed in rabbit and sheep (Marks et al., 1999; Derrick et al., 2007).

**Figure 1:**
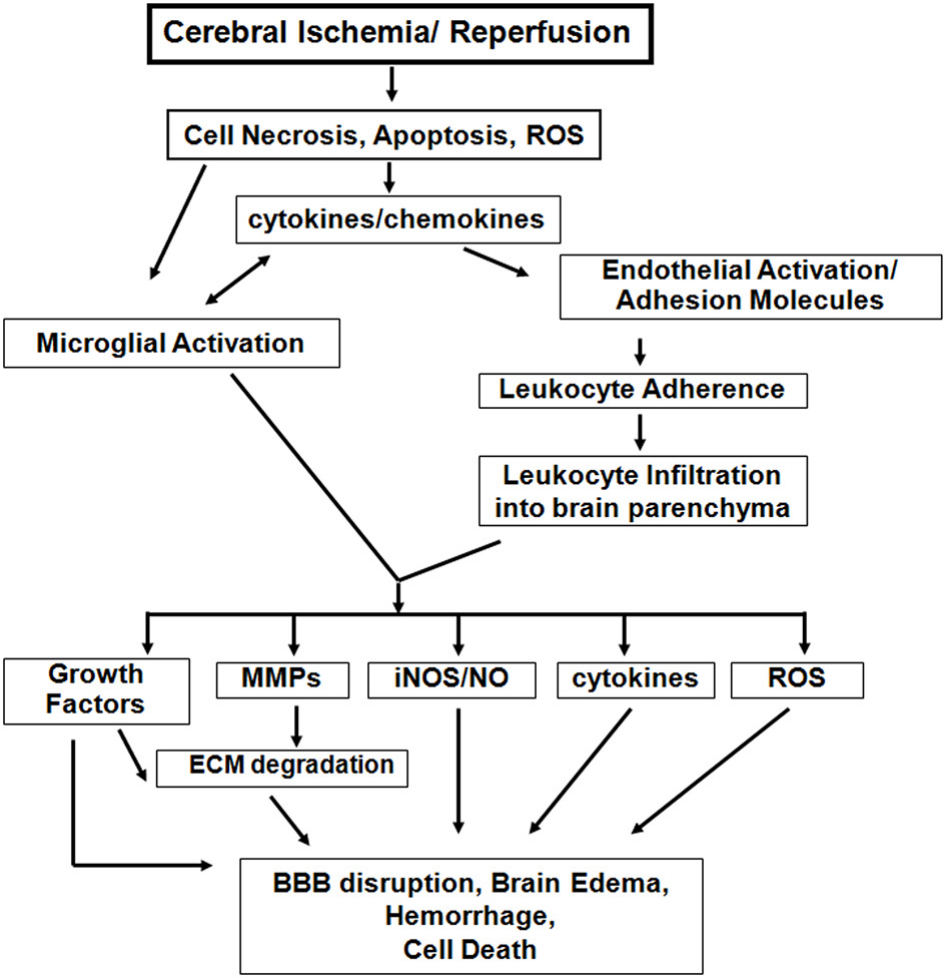
**Inflammatory mechanisms following acute cerebral ischemia-reperfusion injury**. There are both common and distinct features of the inflammatory response to cerebral ischemia between adults and neonates. However, neonatal brain is more susceptible to excitotoxic-damage and oxidative injury by ROS, resulting in necrosis and apoptosis *continuum*. The induction of cytokine/chemokine production, activation of microglial cells, and the systemic inflammatory response lead to neuroinflammation. Differences of adhesion molecule expression on ECs and on peripheral leukocytes exist between the injured adult and neonatal brain. Among the inflammatory mediators, activated matrix metalloproteinases (MMPs), inducible nitric oxide synthase (iNOS), and further cytokine and ROS accumulation also contribute to the variance in magnitude and spatial distribution of BBB disruption, brain edema and injury between neonatal and adult stroke. (modified from Vexler et al., 2006).

Studies in these age-appropriate ischemic models revealed several features unique to neonatal brain injury. First, excitotoxicity-induced neuronal death is a significant injury component (Ikonomidou et al., 1989). Second, neonatal brain is prone to reactive oxygen species (ROS) after HI (Sheldon et al., 2004). Third, apoptosis, a major neuronal cell death mode during this period, coexists with necrosis and necroptosis, creating a “*continuum”* with features of both cell death types (Blomgren et al., 2007; Northington et al., 2011). Finally, inflammation associated with failure to complete apoptosis is pivotal to ischemia-induced injury (Vexler and Yenari, 2009). Figure 1 outlines identified differences in ischemic injury mechanisms between adults and neonates.

## Brain maturation-dependent susceptibility of the BBB after stroke

The BBB undergoes major disruption early after adult stroke. Depending on initial stroke severity BBB opening can be bi-phasic or gradual (Knowland et al., 2014). The timing, extent of leukocyte-mediated BBB disturbances and redistribution of tight junctions (TJs) can vary. Degradation of TJ proteins occludin, claudin-5, and of a TJ-associated protein, zonula occludens 1 (ZO-1), is increased after stroke in part due to activation and/or *de novo* synthesis of MMPs (Yang et al., 2007a; Liu et al., 2012). TJ proteins can also internalize into the cytosol by caveolin-1-mediated endocytosis or redistribute to other membrane domains after cerebral ischemia, coinciding with the early post-ischemic BBB opening. Adherens junction proteins support BBB properties (Petty and Lo, 2002) and their altered composition after stroke changes TJ stability, affecting BBB permeability (Dejana and Giampietro, 2012; Wacker et al., 2012).

**Figure 2:**
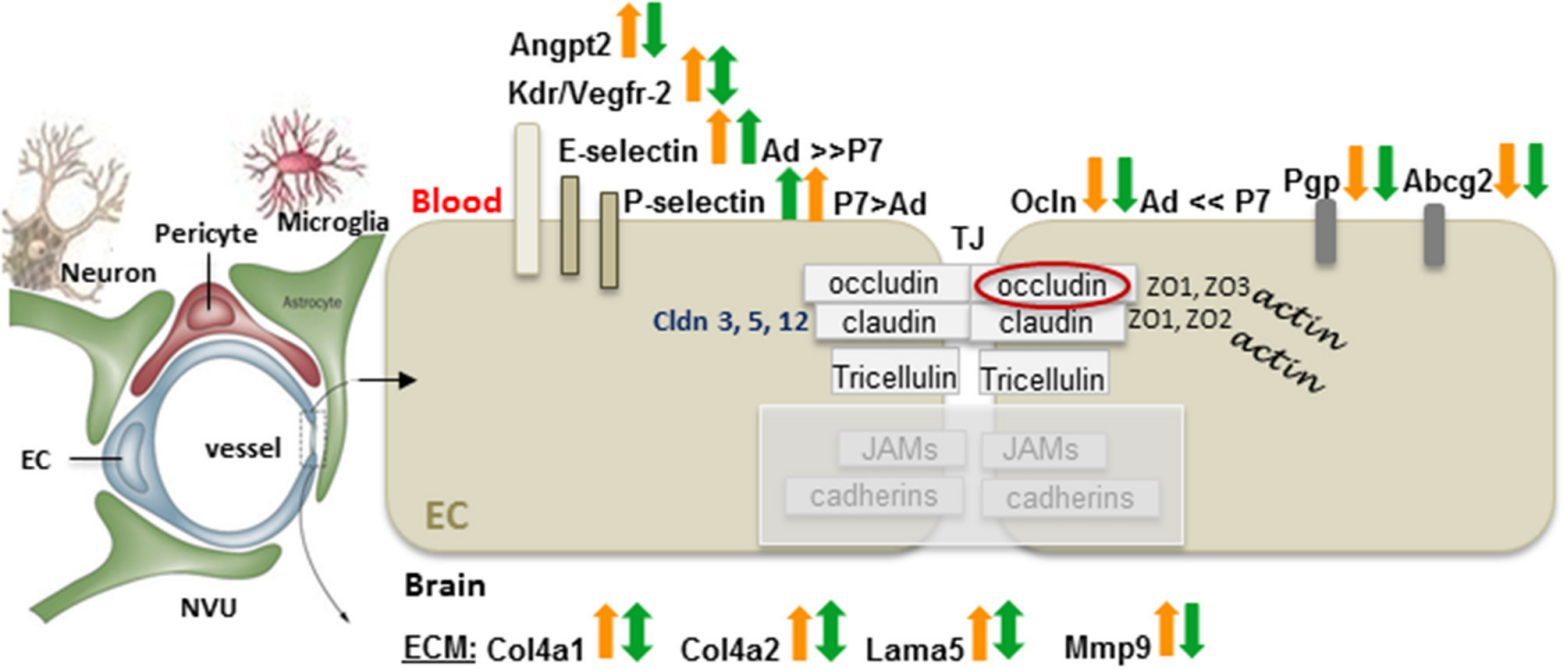
**Differences in the gene expression of the BBB components between adults and neonates 24 h after tMCAO**. On a diagram of the NVU, arrows demarcate the gene expression changes in injured vs. matching contralateral region (ratio, ipsilateral/contralateral) in adult (orange arrows), and neonatal rats (green arrows) subjected to 3 h of MCAO followed by 24 h of reperfusion. Arrows pointing upwards demarcate increased gene expression in injured regions vs. matching contralateral regions (statistically significant > 2-fold change). Downward arrows show reduced expression in injured regions vs. matching contralateral regions (statistically significant > 2-fold change). Double-sided arrows indicate no changes between injured and contralateral regions. Abbreviations: Ad: adult, P7: postnatal 7-days-old, EC, endothelial cell; angiopoetin 2, Angpt2; Kdr, Vegfr-2; Cldn, claudin; Ocln, occludin; ZO, zonula occludens; JAM, junctional adhesion molecules; TJ, tight junction; Pgp, P-glycoprotein; Abcg2, ATP-binding cassette transporter, subfamily G 2; Col4a1, collagen, type IV, alpha 1; Col4a2, collagen, type IV, alpha 2; Lama5, laminin, alpha 5; Mmp-9, matrix metalloproteinase 9. Original data are published in Fernandez-Lopez et al. (2012). The NVU contains fragments from Eichler et al. (2011).

Surprisingly little is known about BBB function after neonatal ischemic injury. Major differences in functional BBB response to acute ischemia-reperfusion between neonates and adults have been recently identified (Fernandez-Lopez et al., 2012). While BBB permeability to albumin or intravascular tracers of a similar size is significantly increased after acute tMCAO in adult rats, BBB permeability remains low in injured neonatal rats. Gene and protein expression of occludin, claudin-5 and ZO-1 are better preserved in injured neonatal brain than in injured adult brain, whereas gene expression of the efflux transporters ATP-binding cassette, subfamily G2 (Abcg2) and P-glycoprotein (P-gp) is reduced in both ages 24 h after reperfusion (Fernandez-Lopez et al., 2012). Transcript levels of several adhesion molecules and ECM components are differentially affected by injury in immature and adult brain, including E-selectin and P-selectin. Gene expression of Mmp-9 is significantly upregulated in injured adults and, while high transcript levels of collagen type IV α1 (Col4a1), and Col4a2 remain unaltered in neonates, a significant increase of these two genes is evident in injured adult rats. Interestingly, transcripts of angiogenic regulators Vegfr-2, and Angpt2 are increased after stroke in adults but not in neonates (Fernandez-Lopez et al., 2012). Figure 2 summarizes these findings. In contrast, a transient leakage of much smaller tracers, sucrose and inulin, was observed in a mouse HI model, with the peak at 6 h and normalization by 24 h (D'Angelo et al., 2012), conveying that size and chemical structure of molecules affect leakage and highlighting the need for future BBB studies in injured immature brain.

The ECM proteins and their corresponding receptors on ECs and astrocytes provide both physical and biochemical glial-vascular “scaffolding” while BM components laminin, collagen IV (Col-IV), fibronectin, and perlecan provide proper cell-cell interactions. Endothelial-ECM interaction via β 1 integrins regulates the expression of claudin-5 and BBB tightness whereas other ECM proteins, like galectin-3 mediate integrin-induced stabilization of focal adhesions, and activate cytokine receptors to enhance actions of growth factors (Goetz et al., 2008). Homozygous mutations in Col-IV are lethal in mid-gestation due to blockage of capillary bed development, and mutations in the COL4A1 gene cause ICH both in newborn mouse and human (Gould et al., 2005; Labelle-Dumais et al., 2011). Laminin degradation after focal stroke in adults causes detachment of astrocytic endfeet, disrupts BBB and induces ICH (Fukuda et al., 2004), while in neonates expression of this ECM protein is not reduced acutely (Fernandez-Lopez et al., 2012). The role of other ECM proteins in injured neonates is less studied but opposite effects of galectin-3 in adult stroke and HI has been demonstrated (Doverhag et al., 2010; Lalancette-Hebert et al., 2012).

## Parenchymal brain cells as modulators of BBB integrity after neonatal stroke

Neuroinflammation is a characteristic feature of stroke progression and a major contributor to brain injury (Iadecola and Anrather, 2011). Multiple cell types (i.e. neurons, astrocytes, ECs, and microglia) increase production of inflammatory mediators post-ischemia, which can adversely affect BBB integrity and propagate injury.

ECs are sensitive to oxidative stress (Freeman and Keller, 2012). Excessive ROS accumulation generated by ischemia/reperfusion contributes to damage of TJs and other EC components, and promotes activation of cell death pathways (Rizzo and Leaver, 2010). Although less sensitive to cerebral ischemia than neurons, ECs also undergo cell death via several mechanisms (Rizzo and Leaver, 2010). Our comparison of gene expression in ECs from injured and contralateral cortex in neonatal and adult rats after tMCAO (endothelial transcriptome) revealed a markedly distinct signature of up-regulated and down-regulated transcripts in injured regions between two ages (Fernandez-Lopez et al., 2012).

Astrocytes are rather resistant to ischemic injury but impairment of water fluxes through astrocytic swelling and increased expression of aquaporin 4 (AQP4) leads to edema after adult focal ischemia (Loreto and Reggio, 2010). No edema was observed in injured P10 pups in brain regions with greatest AQP4 expression (Badaut et al., 2007), suggesting that enhanced water clearance at border regions can protect.

Microglial cells have been considered toxic after cerebral ischemia due to production of inflammatory mediators. However, phagocytotic ability and production of growth factors by microglia can be beneficial via several mechanisms, including support of neuronal and endothelial survival (Baburamani et al., 2012; Luo and Chen, 2012). Microglial cells have several direct modulatory effects on the vasculature. During brain development they mediate vasculogenesis (Arnold and Betsholtz, 2013), and act as a physical bridge that guides vascular anastomosis, facilitating normal angiogenesis, and vascular sprouting. Microglia patrol the vasculature in the naïve brain (Davalos et al., 2012) and, upon BBB disruption, rapidly extend their processes, shielding injured sites (Nimmerjahn et al., 2005). Depletion of microglia worsens parenchymal injury in neonatal rats after tMCAO, increases levels of inflammatory mediators in acutely injured regions, and induces hemorrhagic transformation (Faustino et al., 2011; Fernandez Lopez et al., 2014) perhaps due to differing cell phenotypes in the immature brain.

Pericytes are important regulators of vessel contractility. Pericyte loss or dissociation from vessels lead to edema (Peppiatt et al., 2006) and impaired reflow (Yemisci et al., 2009). Lower than in adult pericyte coverage in the neonatal brain (Daneman et al., 2010) may differentially affect CBF regulation and BBB function after injury but this is yet to be demonstrated.

OPCs support EC survival but they are sensitive to ROS and can adversely affect BBB integrity via release of inflammatory mediators. Mast cells contribute to early ischemic brain swelling, BBB leakage and neutrophil infiltration (Strbian et al., 2006). Their stabilization protects and reduces hemorrhage formation (Strbian et al., 2007). Degranulation of mast cells is injurious in neonatal HI (Jin et al., 2007) and stroke (Biran et al., 2008) but direct effects on BBB have not been studied.

## Systemic inflammation alters BBB permeability after neonatal stroke

Parenchymal, perivascular and peripheral circulating cells independently and in concert contribute to stroke-induced production of inflammatory mediators, upregulation of integrins and adhesion molecules on ECs and leukocytes (Iadecola and Anrather, 2011).

Neutrophils rapidly, often transiently infiltrate ischemic tissue and exacerbate reperfusion injury in adults by priming the endothelium, causing “no-reflow” phenomenon, releasing ROS and proteolytic enzymes, and stimulating cytokine release (Mori et al., 1992; Amantea et al., 2009; Engelhardt and Liebner, 2014). Both loosely attached and infiltrated leukocytes can contribute to generation of pro-inflammatory molecules in the CNS (Denes et al., 2010). Neutropenia or treatments that prevent leukocyte adhesion and infiltration are neuroprotective (Kochanek and Hallenbeck, 1992; Yamasaki et al., 1995). Compared to adults, in neonates neutrophil infiltration is markedly lower in response to HI (Hudome et al., 1997; Bona et al., 1999), and tMCAO (Fernandez-Lopez et al., 2012). Neutralization of cytokine-induced neutrophil chemoattractant 1 protein (CINC-1) following tMCAO in adults holts neutrophil transmigration, reduces brain edema and protects (Yamasaki et al., 1997) whereas it promotes neutrophil infiltration following tMCAO and disrupts the BBB in neonates (Fernandez-Lopez et al., 2012).

Monocytes have been recently shown to have a dual role, elicit both inflammatory effects and maintain NVU integrity following cerebral ischemia. Monocyte depletion, chemokine (C-C motif) receptor 2 (CCR2) knockout, and bone marrow chimeric approach in murine stroke models demonstrated that CCR2 in bone marrow-derived cells alters injury and hemorrhagic transformation (Gliem et al., 2012). The stabilizing effects of monocytes are transforming growth factor beta 1 (TGF-β 1)-dependent (Gliem et al., 2012). Compared to adult stroke, infiltration of circulating monocytes across the BBB is relatively low during the acute injury phase in neonates (Denker et al., 2007). It remains poorly understood whether leukocyte immaturity at the time of insult or a distinct gene expression pattern of selectins, and cytokines/chemokines account for the difference.

T and B cell infiltration may be less profound (Bona et al., 1999) or transient (Benjelloun et al., 1999) in injured neonates than in adults (Catania and Lipton, 1998; Chu et al., 2014).

## Individual inflammatory signaling mechanisms

Evidence is growing that the inflammatory responses after stroke are different in neonates and adults (Vexler and Yenari, 2009). Genetic deletion of various inflammatory mediators, including NADPH oxidase (Doverhag et al., 2008), or interleukin 1 (IL-1) β, IL-1α, or both αβ (Hedtjarn et al., 2005) are not neuroprotective in the neonatal brain compared to adults. Some mediators that are upregulated and injurious during the acute injury phase, such as nitric oxide (NO), MMPs, macrophage inflammatory protein 1 alpha (MIP-1α), monocyte chemoattractant protein 1 (MCP-1), and complement, may be beneficial and mediate repair (Fernandez-Lopez et al., 2014).

MMPs degrade TJ and BM proteins, including collagen, laminin and fibronectin, thereby leading to brain edema, BBB leakage and leukocyte infiltration. MMP upregulation after stroke contributes to ECM breakdown and acute brain damage (Rosenberg et al., 1998; Asahi et al., 2000). MMP-2 and MMP-9, the two most studied MMPs in stroke, play different roles in BBB disruption (Asahi et al., 2001). MMP-3 targets several ECM components, including laminin and proteoglycans, and propagates injury by mediating BBB opening by inflammatory mediators (Cunningham et al., 2005). While activated microglia, macrophages and infiltrating leukocytes are the major sources of MMPs early after injury (Gidday et al., 2005; McColl et al., 2008), over time activated astrocytes and neurons begin producing MMPs, enhancing repair (Zhao et al., 2006). MMP-9 was shown to predict HIE in human newborns (Bednarek et al., 2012). Cerebrovascular ECs from neonates in culture contain more tissue plasminogen activator (t-PA) and gelatinases upon glutamate challenge than adult cells (Omouendze et al., 2013). MMP inhibition is protective after HI (Chen et al., 2009) but long-term MMP inhibition may holt ECM remodeling, as shown in adult stroke (Zhao et al., 2006).

The patterns of monocyte and neutrophil recruitment are cytokine- and chemokine-specific. The multifaceted roles for α, β, and δ classes of chemokines were shown in adult stroke models (Yamasaki et al., 1995). Integrins are central for cell communication within the NVU and for leukocyte recruitment after stroke (Iadecola and Anrather, 2011) but information on the role of integrins in neonatal stroke is scant.

## Blood-CSF barrier (BCSFB)

The choroid plexuses (CPs), forming the BCSFB, are involved in immune cell entry after brain injury (Shechter et al., 2013). CPs express chemokines and support transepithelial trafficking of neutrophils, monocytes and T cells (Szmydynger-Chodobska et al., 2009, 2012; Kunis et al., 2013). Engraftment of CPs in adult stroke models reduced infarct size and improved neurological function, in part via secretion of glial cell line-derived neurotrophic factor (GDNF), brain-derived neurotrophic factor (BDNF), and nerve growth factor (NGF) (Borlongan et al., 2004). CPs have unique functions in the developing brain (Dziegielewska et al., 2001) but their role in protection of the immature brain after stroke is unknown.

## Neurovascular responses and repair after neonatal stroke

Long-term neural repair is less studied after neonatal stroke than after adult stroke. Cell proliferation in the subventricular zone (SVZ) after ischemia is triggered in both adults (Ohab et al., 2006), and neonates (Plane et al., 2004; Yang et al., 2007b). The dynamic changes within the SVZ neurogenic niche permit neuroblast migration into the ischemic striatum (Young et al., 2011) in the adult, where they express phenotypic region-specific mature neuronal markers (Parent et al., 2002), and into peri-infarct striatum after neonatal HI (Plane et al., 2004; Yang et al., 2007b). Niche astrocytes and SVZ microglia are also involved in neuroblast migration (Young et al., 2011). The newborn SVZ contains numerous cell types, including unipotential astrocytes and OPCs as well as bipotential glial progenitors (Levison and Goldman, 1997).

Given the dynamic nature of postnatal brain development one would expect robust repair processes in neonatal stroke but a 1–2 week delay in induction of angiogenesis was shown after tMCAO in P7 and P10 rats (Shimotake et al., 2010; Dzietko et al., 2013; Fernandez-Lopez et al., 2013) despite induction of VEGF (Mu et al., 2003). Another obstacle for endogenous repair is that proliferating cells in the postnatal SVZ differentiate into astrocytes, rather than neurons or oligodendrocytes, and astrogliosis holts the repair (Spadafora et al., 2010; Gonzalez et al., 2013). Changing the neural stem cell fate to route the differentiation into neurons and oligodendrocytes after stroke, for example, with erythropoietin, enhances the repair (Iwai et al., 2010; Gonzalez et al., 2013). Cell based therapies, including mesenchymal stem cells, improve functional outcomes after neonatal HI (Van Velthoven et al., 2010) and arterial stroke (Van Velthoven et al., 2013) by changing the microenvironment and stimulating Angpt1 and VEGF signaling, which amplify angiogenesis and “loosen” the barrier, allowing vessel remodeling and neuroblast migration.

## Translational aspects and future directions

A broad range of therapeutic agents was used in neonatal ischemic brain injury models to target the excitotoxic, oxidative, and inflammatory injury components, but, as in adult stroke, studies revealed limits in protection. To date, hypothermia is the only neuroprotective treatment for perinatal HIE with efficacy limited to moderate injury (Azzopardi et al., 2009; Edwards et al., 2010).

Importantly, recent studies have improved our understanding of the events at the BBB after neonatal ischemia by revealing that the developmental stage of the BBB at the time of ischemic insult is of prime importance and that careful consideration should be given to whether the BBB is in fact disrupted or it limits therapies from reaching an injured neonatal brain. The role of local parenchymal cells, microglia, as modulators of neurovascular integrity after neonatal stroke was also uncovered. Future studies should shed light on relationships between neurovascular integrity and interaction with neuroprogenitors, endogenous, or engrafted, including the migration and differentiation of neural progenitors during stroke-induced neurogenesis.

## Author contributions

Ingrid Kratzer and Sophorn Chip wrote individual parts of the review and contributed equally. Together with Zinaida S. Vexler who provided conceptual framework and wrote parts of the review, they contributed to revisions and prepared artwork.

### Conflict of interest statement

The authors declare that the research was conducted in the absence of any commercial or financial relationships that could be construed as a potential conflict of interest.
